# Remnant cholesterol concentrations best explain the cardiovascular benefit of APOC3 genetic inhibition: a drug target Mendelian randomization study

**DOI:** 10.1093/ehjopen/oeaf018

**Published:** 2025-03-04

**Authors:** Eloi Gagnon, Dipender Gill, Stephen Burgess, Benoit J Arsenault

**Affiliations:** Centre de recherche de l’Institut universitaire de cardiologie et de pneumologie de Québec, Université Laval, Y-3106, 2725 chemin Ste-Foy, Québec, QC, Canada G1V 4G5; Department of Epidemiology and Biostatistics, School of Public Health, Imperial College London, 90 Wood Ln, London W12 0BZ, UK; MRC Biostatistics Unit, University of Cambridge, East Forvie Building, Forvie, Robinson Way, Cambridge CB2 0SR, UK; Cardiovascular Epidemiology Unit, Department of Public Health and Primary Care, University of Cambridge, Cambridge CB2 0SL, UK; Centre de recherche de l’Institut universitaire de cardiologie et de pneumologie de Québec, Université Laval, Y-3106, 2725 chemin Ste-Foy, Québec, QC, Canada G1V 4G5; Department of Medicine, Faculty of Medicine, Université Laval, 1050 Av. de la Médecine, Québec City, QC, Canada G1V 0A6

**Keywords:** Drug target MR, Mendelian randomization, Apolipoprotein C3, Coronary artery disease

## Abstract

**Aims:**

Apolipoprotein C-III (APOC3) inhibitors are approved for hypertriglyceridaemia. Genetic evidence suggests that APOC3 inhibition may also prevent coronary artery disease (CAD), but mechanisms remain unclear.

**Methods and results:**

To clarify how APOC3 inhibition could prevent CAD, we performed two-step cis-Mendelian randomization using genetic variants in the *APOC3* gene region associated with plasma levels of APOC3. For comparison, we investigated proprotein convertase subtilisin/kexin type 9 (PCSK9). Potential mediators included apolipoprotein B, triglycerides, LDL-cholesterol, and remnant cholesterol measured by nuclear magnetic resonance spectroscopy in mostly fasting samples from Karjalainen et al., and in non-fasting samples from the UK Biobank. CAD data were from CARDIoGRAMplusC4D. APOC3 associations with apolipoprotein B and remnant cholesterol levels were two-fold larger in the study by Karjalainen et al. (55% fasted individuals) when compared with the UK Biobank study (non-fasted individuals). Genetically predicted lower APOC3 and PCSK9 levels were similarly associated with reduced CAD risk (OR = 0.83, 95% CI = 0.75–0.92, *P* = 4.6e-04 and 0.76, 95% CI = 0.73–0.80, *P* = 1.6e-31, respectively). In the two-step cis-Mendelian randomization analysis, the association between genetically predicted APOC3 and CAD was attenuated to null when adjusting for apolipoprotein B, triglycerides, or remnant cholesterol. Multivariable Mendelian randomization using genome-wide variants showed that remnant cholesterol, not triglycerides, was conditionally associated with CAD risk.

**Conclusion:**

Remnant cholesterol best explains the mechanism through which APOC3 inhibition could prevent CAD. APOC3 inhibition may influence fasting remnant cholesterol to a greater extent than non-fasting remnant cholesterol. People with high levels of remnant cholesterol could benefit from APOC3 inhibition.

## Graphical abstract

**Graphical Abstract:**
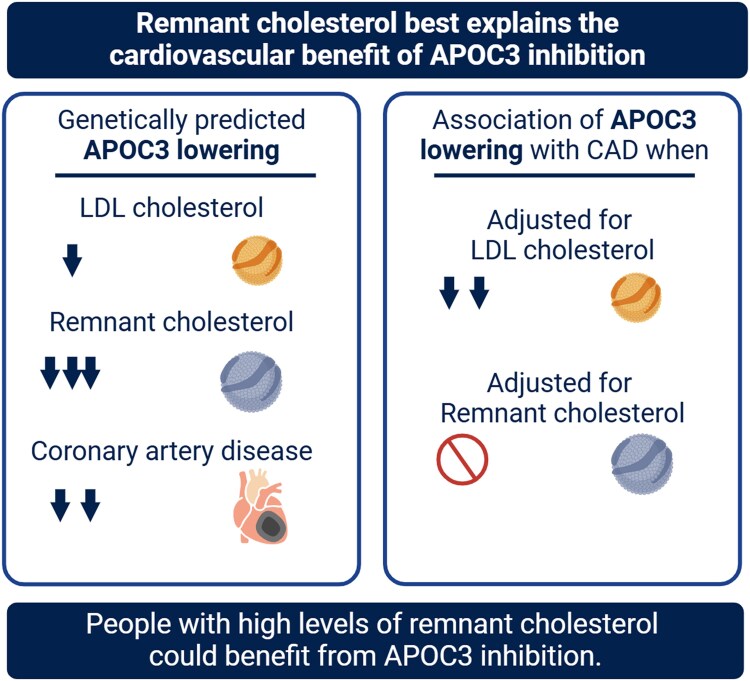

## Introduction

Despite reductions in cardiovascular disease (CVD) event rates at the population level, CVD remain the leading cause of morbidity and mortality worldwide.^1^ The most effective CVD prevention strategies focus on managing hypercholesterolemia, primarily through low-density lipoprotein (LDL) cholesterol-lowering medications. However, some patients continue to face significant cardiovascular risk even with these medications, highlighting the need to develop novel therapies. Drugs targeting apolipoprotein C-III (*APOC3)* have been approved for the management of hypertriglyceridaemia^2–4^ and are now gaining interest as a potential therapy for CVD prevention. This interest is driven by genetic evidence supporting a causal role of APOC3 inhibition in preventing CVD. Indeed, heterozygous carriers of loss-of-function mutations in *APOC3* have a 46% lower risk of coronary heart disease^5^ and 36% lower risk of ischaemic heart disease.^6^ Additionally, our previous Mendelian randomization (MR) analysis showed that genetically predicted lower plasma levels of the APOC3 protein are associated with a significantly reduced risk of coronary artery disease (CAD).^7,8^ However, our understanding of how APOC3 levels could influence CVD is incomplete.

*In vivo* experiments support that APOC3 knockout may prevent atherosclerosis potentially by increasing the clearance of triglyceride-rich lipoproteins and their remnants.^9,10^ This is achieved through the activation of lipoprotein lipase^11^ and increased hepatic clearance of apolipoprotein B (APOB) containing lipoprotein particles.^12^ In humans, APOC3 interference by the small interfering RNA plozasiran led to a 62% decrease in triglyceride levels, a 46% decrease in remnant cholesterol, a 15% lower ApoB, and an 11% lower LDL-cholesterol.^13^

In the Copenhagen City Heart Study, heterozygous carriers of APOC3 inactivating mutations had 43% lower levels of remnant cholesterol and 36% lower risk of ischaemic heart disease, with mediation analysis suggesting that remnant cholesterol could mediate up to 54% of the cardiovascular benefit of APOC3 genetic inactivation.^14^ However, this study did not measure remnant cholesterol directly, but estimated it using the Friedewald Formula introducing measurement error which typically leads to underestimation of the proportion mediated.

Recent large-scale genetic datasets on lipoprotein-lipid levels^15^ can be leveraged to better understand the mechanism of action of APOC3 lowering by identifying lipoprotein-lipid biomarkers of mediation. Mediation can be estimated using the product and difference of coefficient method, through two-step cis-MR.^16^ This approach retains the benefits of using genetic instruments for causal inference, potentially avoiding bias due to confounding, which is a limitation of classic mediation analysis.^17^ In the context of drug target MR, using genetic variants from the gene region of the target of interest limits the issue of pleiotropy and allows for better causal inference.^18^

In this study, we performed drug target MR to assess the association of genetically predicted APOC3 lowering, as well as PCSK9 lowering as a comparator, with lipoproteins-lipid levels and CAD. We then used Two-Step cis-MR to identify lipoprotein-lipid particles acting as biomarkers of mediators. By shedding light on the potential mechanisms of APOC3, we inform drug development efforts targeting the APOC3 protein.

## Methods

### Study exposures and outcomes

This study adheres to the principles outlined in the Declaration of Helsinki. The datasets used for deriving the study exposures and outcomes are detailed in Supplementary material online, *Table S1*. Informed consent was obtained by the respective ethics committee from all individuals who participated in the genome-wide association studies that were included in the analysis. Plasma APOC3 and PCSK9 protein levels were measured in the deCODE cohort, consisting of 35 365 Icelanders.^19^ Protein levels were measured using the SomaScan multiplex aptamer assay (version 4). These measurement levels were adjusted for age and sex and the resulting residuals were inverse rank normal transform prior to genome-wide association study (GWAS). Genotyping was performed using Illumina single nucleotide polymorphisms (SNP) chips. Remnant cholesterol, LDL-cholesterol, HDL-cholesterol, total triglycerides, and apoB levels were quantified by nuclear magnetic resonance spectroscopy in up to 136 016 participants from 33 cohorts of various ancestries.^15^ The majority of the samples were fasted (fasted *n* = 68 559 and non-fasted *n* = 58 112). These measurements were adjusted for age, sex, principal components of ancestries, and relevant study-specific covariates. The resulting residuals were normalized using an inverse rank normal transformation. We also included data on remnant cholesterol, LDL-cholesterol, HDL-cholesterol, total triglycerides, and apoB levels quantified by nuclear magnetic resonance spectroscopy in 114 999 UK Biobank participants of European ancestry.^20^ GWAS was performed using a linear mixed model method as implemented in BOLT-LMM (v2.3) correcting for genotype array, sex, and the ten first genetic principal components. All measures were standardized and normalized prior to analyses using rank-based inverse normal transformation. These summary statistics were accessed through the IEU Open GWAS Project.

Lipoprotein(a) was measured in blood samples of up to 363 228 individuals from the UK Biobank.^21^ Log-transformed measurements were residualized using the top 40 principal component of ancestry, age, sex, self-identified ethnicity, fasting time, estimated sample dilution, assessment centre indicators, genotyping batch indicators, and day of assay. For better comparability, we standardized the unit of measurement to a 1-SD deviation unit using the `coloc::sdY.est` function in the coloc V.5.2.3 R package.^22^ The GWAS for coronary artery disease was a meta-analysis of Biobank Japan, EPIC-CVD, the CARDIoGRAMplusC4D consortium, and other cohorts with 181 522 cases among 1 165 690 participants predominantly of European ancestry.^23^

### Genetic instruments selection and harmonization

The genetic instrument selection strategy varied depending on the exposures (protein vs. lipoprotein-lipid levels) and the type of MR analyses (univariable vs. multivariable MR). For APOC3 and PCSK9 protein levels, we identified common variants (minor allele frequency > 0.01) within a 100-Kb window around the gene region associated (*P* < 5e-8) with lower blood protein levels in the deCODE (*n* = 35 365) population-based cohort. These variants were clumped respectively to the lowest *P*-value (linkage disequilibrium *r^2^* < 0.1 and window = 10 MB) to ensure independence. To prevent epitope binding artefacts, which can occur when an SNP alters the structure of a protein and biases the assessment of blood protein levels, we annotated these SNPs using the variant effect predictor^24^ and removed any SNPs tagged as missense variants, stop gained, stop lost, start gained, start lost, or frameshift. For lipoprotein-lipid levels, we selected common variants (minor allele frequency > 0.01) from throughout the genome robustly (*P* < 5e-8) associated with each lipoprotein-lipid level. Variants were then clumped respectively to the lowest *P*-value of any of these exposures using a more stringent linkage disequilibrium threshold (linkage disequilibrium *r^2^* < 0.01 and window = 1 MB).

The strength of every instrument was evaluated with the Cragg–Donald F-statistic in the univariable MR setting^25^ and by using the conditional *F* statistics in the multivariable MR setting.^26^ Variant harmonization was performed by aligning the betas of different studies on the same effect allele with the *TwoSampleMR* V.0.6.2 package.^27^ When a particular exposure SNP was not present in the outcome dataset, we used proxy SNPs instead (*r^2^* > 0.8). We used the linkge disequilibrium (LD) matrix of the 1000 Genomes Project—European sample of the Utah residents from North and West Europe.

### Mendelian randomization analyses

For univariable MR analyses, we performed the inverse variance weighted (IVW) method with multiplicative random effects.^28^ MR must respect three core assumptions (relevance, independence, and exclusion restriction) for valid causal inference. Violation of these assumptions can occur if the genetic instruments influence several traits on different causal pathways. This phenomenon, referred to as horizontal pleiotropy, can be balanced by applying robust MR methods.^29^ To verify if pleiotropy likely influenced the primary univariable MR results, we performed four different robust MR analyses: the Egger regression,^30^ the contamination mixture,^31^ the weighted median, and the MR-PRESSO,^32^ each making a different assumption about the underlying nature of the pleiotropy. Consistent estimates across these methods provide further confirmation of the nature of the causal relationships. Univariable MR analyses were performed using the *TwoSampleMR* V.0.6.2 package.^27^ To ensure that the results were not driven by the remaining correlation between genetic instruments, we also performed inverse variance weighted adjusted for the linkage disequilibrium matrix using the *MendelianRandomization* V.0.10.1 package.^33^ For multivariable MR analysis, we conducted the IVW method^34^ using the *MendelianRandomization* V.0.10.0 package.^33^

### Mediation analyses

We used the product and difference of coefficient method to perform mediation analysis. We used variants restricted to the vicinity of the gene region of interest and performed Two-Step cis-MR.^16^ Briefly, the variant-outcome association can be decomposed into a path that goes through the mediator and a path independent of the mediator (i.e. the direct path). The direct path was estimated with the product and difference of coefficient method by subtracting the total effect from the indirect effect. The indirect effect was calculated by multiplying the variants-mediator association with a constant being the total effect of the mediator on the outcome, which was estimated using two sample MR with genetic instruments from throughout the genome robustly (*P* < 5e-8) and independently (*r*^2^ < 0.1) associated with the mediator. Standard errors were obtained using bootstrapping with one million iterations.

## Results

We identified 8 genetic instruments explaining 1.8% of the variance of the circulating levels of APOC3, with a mean *F*-statistics of 82, indicating adequate instrument strength. The genetic instruments for PCSK9 were similarly strong (*r*^2^ = 0.04 and mean *F*-statistics = 102). All estimates are expressed per 1 SD lower plasma levels of either APOC3 or PCSK9 to reflect the potential effect of drugs targeting their circulating levels.

**Figure 1:**
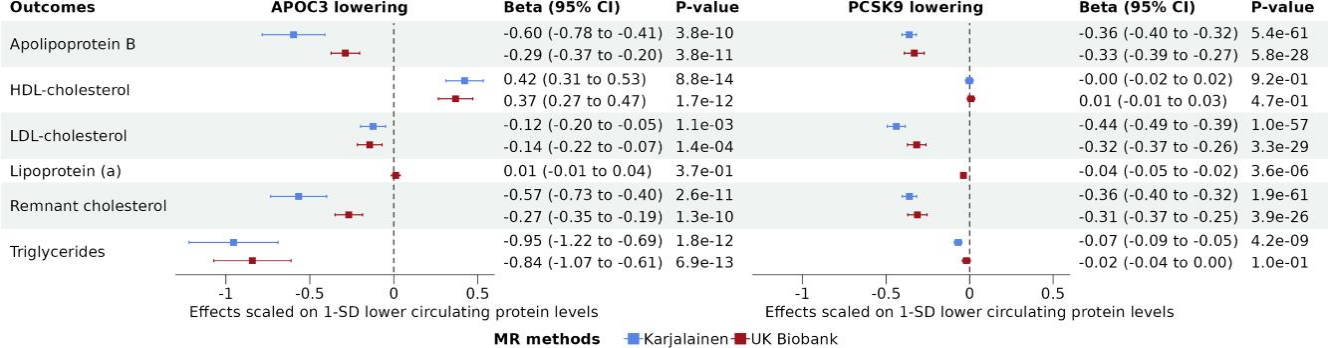
Association between 1-SD lower genetically predicted APOC3 and PCSK9 plasma levels and lipoprotein-lipid levels. The different colours represent the different datasets. Genetically predicted APOC3 and PCSK9 were proxied using 8 and 14 cis genetic instruments, respectively. Estimates and confidence intervals were obtained with random effect inverse variance weighted Mendelian randomization.

Genetically predicted reduction in APOC3 and PCSK9 showed distinct association profiles with lipoprotein-lipid levels (*Figure 1*). Using data from Karjalainen et al., lower levels of APOC3 were associated with a nearly two-fold stronger decrease in remnant cholesterol (−0.57 95% CI = -0.73 to −0.40, *P* = 2.6e-11) compared to PCSK9 (−0.36 95% CI = −0.40 to −0.32, *P* = 1.9e-61) and a two-fold stronger decrease in apoB (−0.60 95% CI = −0.78 to −0.41, *P* = 3.8e-10) compared to PCSK9 (−0.36 95% CI = −0.40 to −0.32, *P* = 5.4e-61). The APOC3 association with apoB and remnant cholesterol levels was twice as strong in the study by Karjalainen et al., when compared with the UK Biobank study, while the PCSK9 associations with apoB and remnant cholesterol were similar across the studies. Lower levels of PCSK9 were associated with a nearly fourfold stronger decrease in LDL-C (−0.44 95% CI = −0.49 to −0.39, *P* = 1.0e-57), when compared to APOC3 (−0.12 95% CI = −0.20 to −0.05, *P* = 1.1e-03) in both studies. Lower PCSK9 levels were associated with a slight decrease in lipoprotein(a) (−0.04 95% CI = −0.05 to −0.02, *P* = 3.6e-06), while lower levels of APOC3 were not associated with lipoprotein(a). Lower levels of APOC3 were associated with an increase in HDL-cholesterol (0.42 95% CI = 0.31–0.53, *P* = 8.8e-14), but lower levels of PCSK9 were not associated with HDL-cholesterol levels.

**Figure 2:**
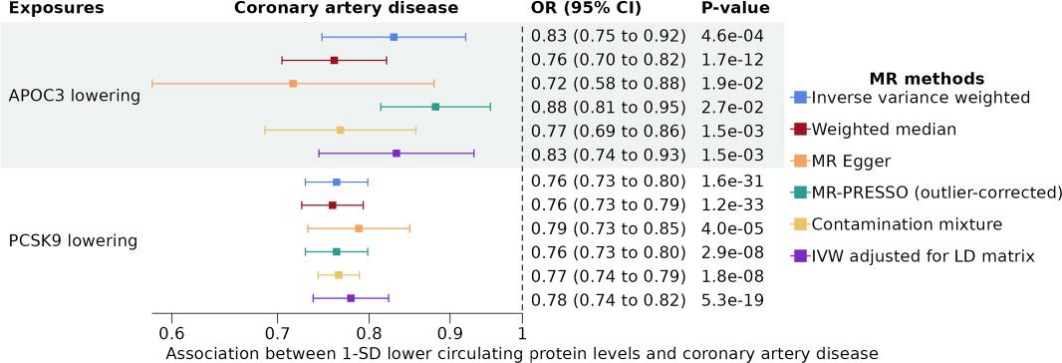
Association between 1-SD lower genetically predicted APOC3 and PCSK9 plasma levels and coronary artery disease. The different colours represent the different MR methods used. Genetically predicted APOC3 and PCSK9 were proxied using 8 and 14 cis genetic instruments, respectively. Estimates and confidence intervals were obtained with random effect inverse variance weighted Mendelian randomization.

Genetically predicted APOC3 lowering was associated with a lower risk of CAD (odds ratio [OR] per 1 SD [standard deviation] lower APOC3 plasma levels = 0.83, 95% CI = 0.75–0.92, *P* = 4.6e-04) (*Figure 2*). Each standard deviation decrease of genetically predicted PCSK9 levels was similarly associated with a lower risk of CAD (OR = 0.76, 95% CI = 0.73–0.80, *P* = 1.6e-3). These results were directionally consistent and maintained statistical significance (*P*-value < 0.05) across all pleiotropy robust MR methods (weighted median, MR Egger, contamination mixture, MR-PRESSO) and when accounting for linkage disequilibrium.

### Identifying the mediators of APOC3 inhibition and CAD

**Figure 3:**
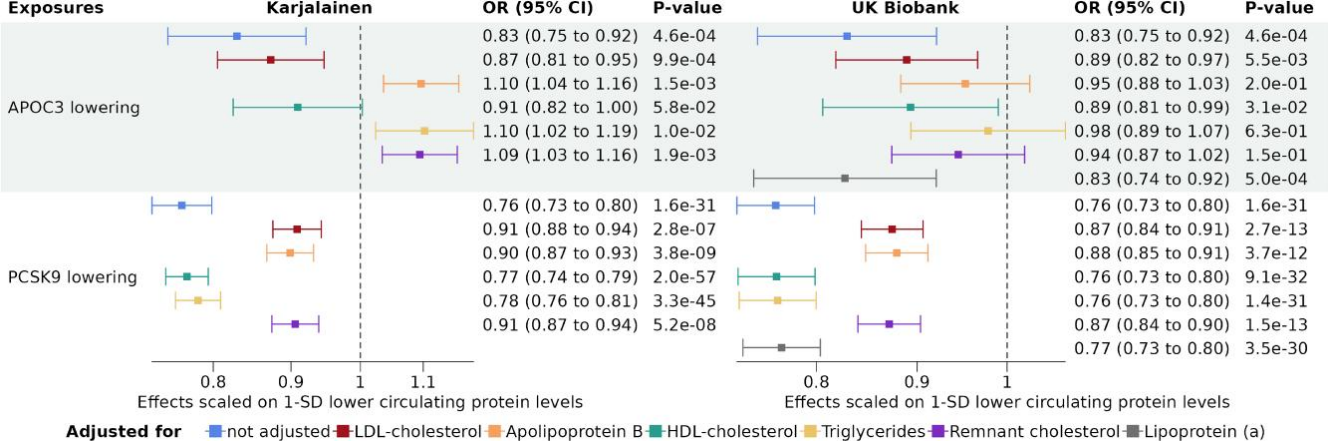
Association between 1-SD lower genetically predicted APOC3 and PCSK9 plasma levels and coronary artery disease with and without adjustment for lipoprotein-lipid particles with two-step cis-MR method. The different colours represent the different lipoprotein-lipid traits. Genetically predicted APOC3 and PCSK9 were proxied using 8 and 14 cis genetic instruments, respectively. Estimates and confidence intervals were obtained with random effect inverse variance weighted Mendelian randomization.

To identify potential lipoprotein-lipid parameters that may be biomarkers of mediating mechanisms for the association between APOC3 levels and CAD, we performed Two-Step cis-MR.^16^ We included the same cis-acting genetic instruments of APOC3 and PCSK9 as in the previous univariable MR. We calculated the mediator-CAD association using two sample MR with genetic instruments from throughout the genome robustly (*P* < 5e-8) and independently (*r*^2^ < 0.01) associated with the mediator. The association of PCSK9 levels and CAD did not change when adjusting for lipoprotein(a), HDL-cholesterol, or triglyceride levels, but was significantly lowered when adjusting for APOB, LDL-cholesterol, or remnant cholesterol (*Figure 3*). In contrast, for APOC3, the main biomarkers of mediation were apoB and triglyceride-rich lipoprotein concentration. Indeed, the association of APOC3 lowering and CAD did not change much when adjusting for lipoprotein(a), HDL-cholesterol, or LDL-cholesterol levels, but was significantly lowered when adjusting for apoB, remnant cholesterol or triglyceride levels. Using UK Biobank data, we estimated that 69% (95% CI = 25–114) of the effect of APOC3 on CAD could be mediated by remnant cholesterol.

**Figure 4:**
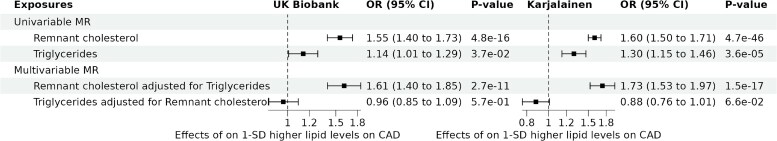
Association between 1-SD higher genetically predicted remnant cholesterol and triglyceride plasma levels and coronary artery disease, with and without adjustment for each other in multivariable MR. The estimates under the univariable MR section are unadjusted estimates, whereas the estimates under the multivariable MR section are adjusted. Estimates and confidence intervals were obtained with random effect inverse variance weighted Mendelian randomization and multivariable inverse variance weighted Mendelian randomization.

Given that triglyceride levels and remnant-cholesterol levels are highly correlated we tested the independent association of triglycerides and remnant cholesterol with CAD using genetic variants from throughout the genome in a multivariable MR analysis. For univariable MR analysis, we included as instruments all independent SNPs (*r*^2^ < 0.01) robustly (*P*-value < 5e-8) associated with triglycerides (to instrument triglycerides) and remnant cholesterol (to instrument remnant cholesterol). For multivariable MR, we included these SNPs and clumped them (*r*^2^ < 0.01) respective to the lowest *P*-value of any of these traits. In univariable MR, both remnant-cholesterol and triglyceride levels were associated with an increased risk of CAD (*Figure 4*). In multivariable MR, only remnant cholesterol remained significantly associated with CAD, while the association of triglycerides reverted and became null. These results support that remnant cholesterol could be the main mediator.

## Discussion

To understand the mechanisms through which therapies targeting APOC3 could prevent CAD, this multivariable MR study evaluated the association of genetically predicted APOC3, using PCSK9 as a comparator, with CAD and lipoprotein-lipid particle parameters as biomarkers of mediation. Genetically predicted APOC3 was associated with reduced CAD risk, but this association attenuated when accounting for apoB, triglycerides, or remnant cholesterol. Multivariable MR evaluating the mutually adjusted associations of remnant cholesterol and triglycerides with CAD favoured remnant cholesterol as the main risk factor. Although this remains to be tested in human trials, these results provide genetic evidence that remnant cholesterol may explain why APOC3 inhibitors could prevent CAD.

Genetic associations of *APOC3* variants align with the results of the most recent trial on plozasiran, a small interfering RNA targeting liver APOC3 synthesis, thus validating our genetic instruments. In a recent phase 2b, double-blind, randomized, placebo-controlled trial, plozasiran was administered every 12 weeks to patients with mixed hyperlipidemia.^13^ After 24 weeks, participants in the plozasiran arm exhibited 60% lower APOC3 levels, 62% lower triglyceride levels, 46% lower remnant cholesterol, 15% lower ApoB, 11% lower LDL-cholesterol, 30% higher HDL-cholesterol, and no difference in lipoprotein(a) when compared to the control group.^13^ The genetic associations show a similar pattern of associations with lipoprotein-lipids levels. These concordant results provide external validation for our APOC3 genetic instrumentation.

Our results indicate that remnant cholesterol explained most of the association between genetically predicted APOC3 lowering and CAD. This finding aligns with a meta-analysis of 137 895 individuals including 766 heterozygotes carriers of loss-of-function mutation in APOC3.^14^ Carriers exhibited a 41% lower risk of ischaemic vascular disease, 43% lower remnant cholesterol, and 4% lower LDL-cholesterol. Mediation analysis in that study showed that remnant cholesterol explained 37% of the lower risk observed in carriers.^14^ In that study, remnant cholesterol was estimated using the Friedewald Formula (i.e. triglycerides divided by five), and only in one cohort was it measured directly.^14^ This approach was therefore unable to disentangle the mediating effect of triglycerides and remnant cholesterol. As triglycerides do not accumulate in the atherosclerotic plaque, they are unlikely to directly cause atherosclerosis.^35^ Triglycerides likely represent a biomarker of high remnant cholesterol as they are highly correlated (*r* = 0.83).^14^In contrast, our study used nuclear magnetic resonance to directly measure remnant cholesterol, confirming that remnant cholesterol, not triglycerides, is the key mediator. Moreover, the authors estimated associations of the proxied remnant cholesterol using traditional epidemiology, which is prone to confounding. By contrast, our approach used genetic instruments to estimate the causal effect of remnant cholesterol on CAD, mitigating bias due to confounding. Our genetic data clarify the mechanism for the potential cardioprotective effect of ApoC3 inhibitors, highlighting remnant cholesterol as the primary mediator.

Results of our study suggest that patients with high levels of triglycerides or remnant cholesterol could benefit from APOC3 inhibition. A large proportion of patients still experience clinical events despite aggressive lipid-lowering treatments.^36^ This residual risk could be attributed to other risk factors not adequately addressed by licenced therapies. Remnant cholesterol may be one of the most significant and independent contributors to CVD risk after LDL-cholesterol.^37^ Remnant cholesterol can be taken up by the arterial wall and accumulate in the intima and smooth muscle layers leading to foam cell formation and atherogenesis.^38^ MR studies have shown an independent effect of remnant cholesterol, even after adjustment for LDL-C.^39,40^ The mean non-fasting remnant cholesterol in 119 180 UK Biobank participants is 27.79 mg/dL with an SD of 7.36 mg/dL. Assuming this standard deviation in our study, each 1 mg/dL decrease in remnant cholesterol through lifelong genetically predicted APOC3 inhibition was associated with a 5% lower odds of CAD (OR, 0.95; 95% CI = 0.94–0.96, *P* = 4.3e-17). Keeping in mind that MR estimates reflect lifelong differences, and thus are typically larger than trial estimates, Olezarsen resulted in a 30.8 mg/dL placebo-adjusted decrease in fasting remnant cholesterol, which is encouraging.^4^

The fact that the genetically predicted association between APOC3 levels and CAD is primarily explained by remnant cholesterol suggests that remnant cholesterol could serve as an early biomarker to predict the cardiovascular efficacy of APOC3 therapies. Biomarkers are increasingly being included in early-phase clinical trials.^41^ A good biomarker must predict target engagement and future efficacy.^41^ Remnant cholesterol satisfies both criteria as its level is influenced by APOC3 and predicts (and could be causal for) future CAD events. Plozasiran, a small interfering RNA against APOC3, was associated with 46% lower remnant-C when compared to placebo.^13^ This early reduction in remnant cholesterol suggests that pharmacological APOC3-lowering could provide a reduction of cardiovascular events in cardiovascular outcome trial trials. If substantiated by cardiovascular outcome trials, remnant cholesterol could become a screening tool to identify individuals who could benefit from APOC3 inhibition.

In our study, the association between APOC3 and remnant cholesterol was much stronger using data from Karjalainen et al., than from the UK Biobank. This difference could be explained by the different fasting status of the study participants. UK Biobank participants were non-fasting^20^ whereas 55% of participants in the study by Karjalainen et al. were fasting.^15^ During fasting, APOC3 inhibits the activity of lipoprotein lipase which slows the clearance of triglycerides and remnant cholesterol particles from the blood.^12^ This suggests that the effect of APOC3 on lipid levels may be more pronounced in the fasting state, which can help explain why the mediating effect of remnant cholesterol was stronger in the Karjalainen study compared to the UK Biobank. This implies that the lipid-lowering of plozasiran and olezarsen, which was quantified in the fasting state,^4,13^ may not translate to the same lipid lowering in the non-fasting state. Data on lipid-lowering in the non-fasted state could also be informative to predict future cardiovascular efficacy.

The main strength of the present study is the use of an MR design with multiple robust and independent cis-acting instruments, minimizing confounding that often affects traditional mediation analysis. Another strength is the use of the largest genetic association data on directly measured remnant cholesterol. However, our findings must be considered with three key limitations. MR evaluates the lifelong effect of a genetically perturbed target, whereas clinical trials often begin later in life and are of shorter duration. As a result, MR typically estimates stronger effects compared to randomized control trials. Therefore, although MR can inform the design of trials, these MR data are not directly transferable to estimate the magnitude of risk reductions for a CVD outcomes trial. Second, although the GWAS included individuals from mixed ancestry, the majority of the study sample was from European ancestry, which may limit generalizability of the study findings to other ancestries. Third, as Mendelian randomization assumes a linear effect, the current analysis could not find a specific threshold for increased risk.

In conclusion, these results show that remnant cholesterol most closely explains the association between genetically predicted levels of APOC3 and CAD. This suggests that APOC3-lowering therapies could be primarily effective in patients with high baseline levels of triglyceride-rich lipoprotein particles. Thus, APOC3 inhibitors have the potential to be transformative for patients with remnant cholesterol-mediated atherosclerotic diseases.

## Supplementary Material

oeaf018_Supplementary_Data

## Data Availability

All the data used in this study are in the public domain. Supplementary material online, *Table S1* describes the data used and relevant information to retrieve the summary statistics. Code to reproduce the results of this manuscript is available at https://github.com/LaboArsenault/apoc3moa.
